# Dimensions of Psychological Resilience Among Mental Health Professionals in Greece: A Postdoctoral-based Literature Review

**DOI:** 10.1192/j.eurpsy.2024.1404

**Published:** 2024-08-27

**Authors:** A. Kalmanti, N. Smyrnis, I. Michopoulos, V. Yotsidi, V. Moraiti, G. N. Porfyri

**Affiliations:** ^1^2nd Department of Psychiatry, Medical School, National and Kapodistrian University of Athens, “Attikon” University Hospital; ^2^Psychology Department, Panteion University of Social and Political Sciences; ^3^Early Intervention in Psychosis (EIP), Ikelos NGO (Collab. “Attikon” University Hospital), Athens; ^4^Psychiatric Department, General Hospital of Papanikolaou, Thessaloniki, Greece

## Abstract

**Introduction:**

Resilience is defined as the process and outcome of successfully adapting to difficult or challenging life experiences, and adjustment to external and internal demands, including challenges in family or relationship dynamics, serious health concerns, financial pressure or work-related stress. Employees’ creative self-sufficiency, work environment, as well as the interpersonal relationships developing in the workplace which constitute basic parameters of professional satisfaction can potentially affect both psychosomatic resilience of the employees as well as their performance at work. Exploring the available bibliography, it was revealed that the mental health professionals’ community has not been sufficiently examined in terms of emotional resilience.

**Objectives:**

To examine the dimensions of psychological resilience among mental health professionals.

**Methods:**

In the context of a postdoctoral research which is conducted on a sample of the Greek population- personnel working in mental health hospital and community-based settings -a review of 35 articles from 1985 to 2023 on PubMed and Google Scholar was proceeded regarding psychological resilience among mental health professionals.

**Results:**

Creative self-sufficiency and professional satisfaction were found to be positively correlated with resilience among mental health professionals. Additional factors have been found to influence mental resilience among mental health professionals, such as individual personality traits, coping style, perceived social support, a sense of security, and organizational support.

**Conclusions:**

This review contributes to the evolving understanding of resilience, particularly regarding mental health prοviders. The positive correlation between creative self-sufficiency and professional satisfaction highlights the importance of fostering these dimensions to enhance mental resilience through implementing emotional capacity-building practices, social skills counseling, as well as mindfulness-based interventions.

**Disclosure of Interest:**

None Declared

